# Persistence of DNA in Carcasses, Slime and Avian Feces May Affect Interpretation of Environmental DNA Data

**DOI:** 10.1371/journal.pone.0113346

**Published:** 2014-11-17

**Authors:** Christopher M. Merkes, S. Grace McCalla, Nathan R. Jensen, Mark P. Gaikowski, Jon J. Amberg

**Affiliations:** 1 IAP Worldwide Services Inc., Upper Midwest Environmental Sciences Center, La Crosse, Wisconsin, United States of America; 2 United States Geological Survey, Upper Midwest Environmental Sciences Center, La Crosse, Wisconsin, United States of America; University of Houston, United States of America

## Abstract

The prevention of non-indigenous aquatic invasive species spreading into new areas is a goal of many resource managers. New techniques have been developed to survey for species that are difficult to capture with conventional gears that involve the detection of their DNA in water samples (eDNA). This technique is currently used to track the invasion of bigheaded carps (silver carp and bighead carp; *Hypophthalmichthys molitrix* and *H. nobilis*) in the Chicago Area Waterway System and Upper Mississippi River. In both systems DNA has been detected from silver carp without the capture of a live fish, which has led to some uncertainty about the source of the DNA. The potential contribution to eDNA by vectors and fomites has not been explored. Because barges move from areas with a high abundance of bigheaded carps to areas monitored for the potential presence of silver carp, we used juvenile silver carp to simulate the barge transport of dead bigheaded carp carcasses, slime residue, and predator feces to determine the potential of these sources to supply DNA to uninhabited waters where it could be detected and misinterpreted as indicative of the presence of live bigheaded carp. Our results indicate that all three vectors are feasible sources of detectable eDNA for at least one month after their deposition. This suggests that current monitoring programs must consider alternative vectors of DNA in the environment and consider alternative strategies to minimize the detection of DNA not directly released from live bigheaded carps.

## Introduction

Aquatic invasive species (AIS) can have severe ecological and economic effects, and much effort has been put into the control of such species (reviewed in [1]). A key element to controlling AIS is detection of the species while at low abundance and early in the invasion [1]–[3]. Unfortunately, some AIS are difficult to capture using conventional methodologies [4], [5]. Molecular tools, such as the analysis of environmental DNA (eDNA), have been used to quantify populations of elusive animals, identify sex distribution of animals in an area, assess biodiversity, and identify predators of vulnerable species [6]–[16]. The detection of eDNA in aquatic systems has begun to be used to monitor rare fish species, including AIS [4], [17]–[20], reviewed in [21]. This approach can be a very effective and efficient monitoring tool for detecting species at very low population densities, which is imperative for management.

The detection of eDNA has been implemented by resource agencies to monitor for the presence of bighead carp (*Hypophthalmichthys nobilis*) and silver carp (*H. molitrix*) in the Chicago Area Waterway System (CAWS), which connects carp-infested waters of the Mississippi and Illinois rivers to the Great Lakes. Silver carp and bighead carp are closely related species and are known to frequently hybridize [22]. These two species occur together in the Illinois and Mississippi rivers and are referred to as bigheaded carps. To deter bigheaded carps from entering the Great Lakes, electric fish dispersal barriers have been constructed in the Chicago Sanitary and Shipping Canal (CSSC) near Romeoville, IL. The DNA of silver carp has been detected upstream of these electric barriers, but no silver carp has ever been captured above these electric barriers. A single bighead carp was captured in Lake Calumet during routine sampling efforts in June of 2010, but until autumn of 2012, no bighead carp DNA had ever been detected [4], [23]. Silver carp DNA was detected in 18 out of 209 samples collected from the CAWS on 19-June, 2013 [24].

The lack of correlation between DNA detections and the capture of a silver carp increases the importance of evaluating different pathways of DNA entry into the environment. Vectors and fomites may play a significant role in transferring DNA to areas lacking live fish. Barges are one such fomite. These barges travel from heavily silver carp-infested waters to above the electric fish dispersal barrier where no silver carp has been captured but silver carp DNA has been detected. Silver carp have been observed jumping and hitting the sides of barges and even landing and dying on the decks of barges [25], [26]. Additionally, piscivorous birds have been observed defecating on barge decks while docked in bigheaded carp-infested waters, and DNA collected from scats has been used to analyze predator diets (reviewed in [27], [28]). Depending upon the persistence of DNA in these sources, barge transport may be a feasible path through which bigheaded carp DNA can be transferred into bigheaded carp-free waters.

The persistence of DNA in slime, carcasses and bird feces on hard substrates is not well understood and has a direct impact on the risk of DNA transfer by shipping barges. We determined the persistence of DNA from carcasses, slime and bird excrement adhering to simulated barge surfaces and DNA shedding rates from carcasses under environmental conditions.

## Methods

### Ethics Statement

Juvenile silver carp (*Hypophthalmichthys molitrix*) were obtained from stocks held at the United States Geological Survey Upper Midwest Environmental Sciences Center (UMESC), La Crosse, WI. Silver carp were euthanized by an overdose of tricaine methanesulfonate (FINQUEL, Argent Chemical Laboratories, Redmond, WA) then stored at −20°C until used. Procedures for handling and euthanasia of test animals were approved by the UMESC Animal Care and Use Committee (Approval #: AEH-11-CARPODF-01). Procedures for feeding and collecting fecal material from eagles were reviewed and approved by the director of eagle care and consulting veterinarian at the National Eagle Center (NEC); Wabasha, MN (Approval #: AEH-12-EDNA-02). Any use of trade, product, or company name is for descriptive purposes only and does not imply endorsement by the U.S. Government.

### Persistence of DNA

To simulate the occurrence of silver carp carcasses on a barge, a single juvenile silver carp carcass (79.50±4.35 g; mean ± 1 standard deviation) was placed onto each of four non-stick coated steel trays. Trays were separately floated on foam blocks in a caged (1″ square mesh) 0.01 acre pond allowing exposure to natural light regimes and temperature. Each tray was covered by a clear plastic container to prevent carcass loss during a major rain event.

To simulate bigheaded carp slime deposition on a barge, slime of silver carp was accumulated on separate but identical metal trays by placing the carcasses of 10 silver carp on each of four metal trays for one hour before they were removed and discarded. Contact with fish left residual slime mass of 4.15±1.26 g (mean ± 1 standard deviation) on each metal tray which was then handled as described above. Triplicate DNA samples were collected from each carcass and each slime deposit by gently wiping the surface with a swab. DNA samples were collected over the course of 28 days (Days 1, 2, 3, 4, 6, 8, 10, 12, 15, 17, 19 and 28). All samples were stored in 2- mL centrifuge tubes at −80°C until DNA extraction.

To determine the persistence of DNA in bird feces, four eagles (*Haliaeetus leucocephalus*) at the NEC had their silver carp-free diet replaced for a single day (June 25, 2012) with silver carp. Approximately all of the fecal material excreted during the 24 hours following the meal of silver carp was collected from each individual bird. The collected fecal material was immediately placed on wet ice and transported to UMESC where it was split into 8–10 subsamples of 1–2 g. Two subsamples from each eagle were placed on separate convex metal trays and two were placed on separate concave metal trays. The remaining unused subsamples were stored at −80°C. Concave trays were used to ensure that the fecal material stayed on the tray, whereas the convex sheets allowed easy collection of runoff water during simulated rain events. All metal trays were floated on foam blocks in a 0.01 acre caged pond and covered with a clear plastic container to protect from rain but allow for natural light regimes. To examine the persistence of detectable silver carp DNA in those feces, DNA samples were collected at midday daily for 18 days from each metal tray and then again on day 30. The surface temperature of the metal trays and the pond water were measured using an infrared thermometer concurrent with sample collection. From fecal samples on the concave trays, DNA samples were collected by gently rubbing swabs across the fecal material in triplicate. Swabs were individually placed in a 2-mL centrifuge tube and stored at −80°C until DNA extraction. Concurrently, to assess the potential for contained silver carp DNA to be washed from the feces during a rain event, fecal samples from half of the convex sheets were gently sprayed with ∼1.6 mL of deionized water and the other half with equal volume of pond water which is the equivalent of a 2.5-mm rain event. The runoff from each convex sheet was collected and divided into three ∼0.5-mL aliquots. Each aliquot was centrifuged at 5,000× g for 30 minutes then the supernatant was decanted and the resulting pellet stored at −80°C until DNA extraction. It was noted that some samples, particularly those receiving simulated rain, had no visible fecal material remaining by day 30.

### Shedding of DNA from carcasses in water

**Figure 1:**
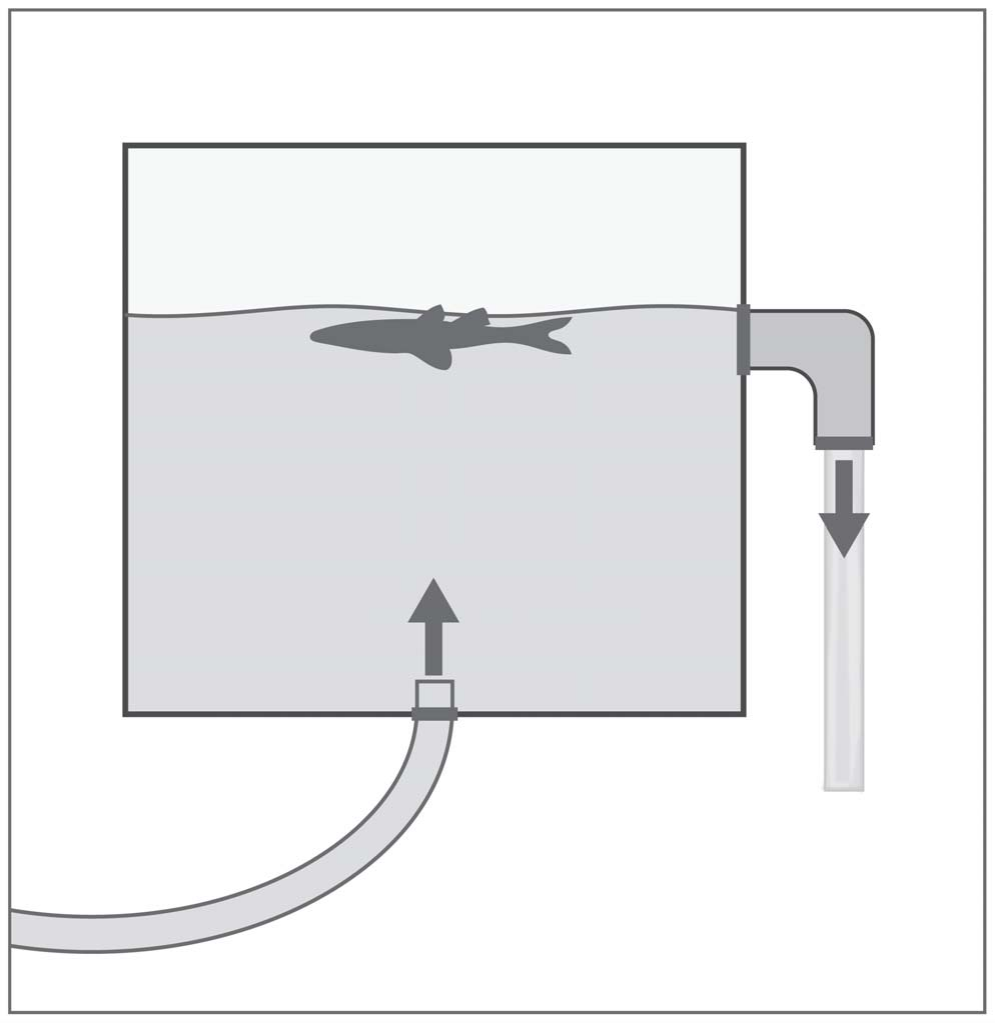
Chambers designed to assess the degradation of DNA from a silver carp carcass. Arrows indicate the direction of water flow.

One or 10 juvenile silver carp carcasses were randomly distributed to 8 flow-through chambers (1L; clear PVC), resulting in 4 chambers containing 97.7 g (±6.7 g standard deviation) of silver carp (single carcass) and 4 chambers containing 902.6 g (±42.7 g standard deviation) of silver carp (10 carcasses). The flow-through chambers were supplied with well water (12–13°C) at a rate of 0.3 L min^−1^ (Figure 1) which turned over the chamber volume >3 times per minute to simulate a fast-flowing stream. Each chamber was placed in a mesocosm as described above. Triplicate water samples were collected from the outflow of each chamber in sterile 50-mL conical tubes, resulting in 12 samples taken per condition of one or 10 carcasses. Spigots occasionally clogged with debris (e.g. microbial growth); clogged spigots were cleared and water was allowed to run through the spigot for >1 minute before taking a sample. Tanks were not covered so that clogged spigots did not stop water flow through the tank (i.e., the same volume of water flowed around the carcass[es]). Immediately following sampling, water samples were centrifuged at 5,000× g for 30 minutes, the supernatant decanted and the resulting pellet stored at −80°C until DNA extraction.

### DNA extraction and detection

DNA was extracted using a commercial extraction kit (DNeasy Blood & Tissue; Qiagen Inc., Valencia, CA) following the manufacturer's protocols with final elution volumes of 200 µL. Conventional PCR was carried out using silver carp-specific primers described in [4] and currently used for monitoring silver carp in the CAWS by the U.S. Fish & Wildlife Service (forward-CCTGARAAAAGARKTRTTCCACTATAA; reverse-GCCAAATGCAAGTAATAGTTCATTC). Thermal cycling conditions were: 94°C for 10 minutes followed by 45 cycles of 94°C for 1 minute, 50°C for 1 minute, 72°C for 1.5 minutes, and a final extension at 72°C for 10 minutes before holding at 4°C [4]. The PCR was performed using an Eppendorf MasterCycler Realplex 2 thermalcycler (Eppendorf NA, Hauppauge, NY). Reactions (20 µL) contained 8 µL of template DNA, 1 µM forward and reverse primers, and 10 µL of 2× MangoMix (Bioline USA Inc., Taunton, MA). Each PCR was done in duplicate, and each plate had additional PCR negative (no template) and positive (silver carp fin extracted DNA) controls. Amplicon presence was verified by agarose gel electrophoresis using a 2% agarose gel, stained with GelRed (Biotium Inc., Hayward, CA), visualized under UV light on a BioDoc-It imaging system (UVP, LLC, Upland, CA). Samples from the carcass and slime experiments were processed starting with Day 1, then Day 8. After that, samples were processed in consecutive and reverse order from Day 8 to find the first day with no detections and the last day that all samples detected silver carp DNA. Because all samples on Days 1 and 4 detected positive, samples on Days 2 and 3 were assumed to be positive. Samples from the eagle feces were processed starting with Day 18. Because all samples on Day 18 detected positive, all samples on prior days were assumed to be positive, and all samples on Day 15 were processed to confirm. Samples on Day 30 were processed afterward.

Quantitative PCR was completed on water samples from days 1, 6, 17 and 28 using silver carp-specific primers (forward-GGTGGCGCAGAATGAACTA; reverse-TCACATCATTTAACCAGATGCC) and a silver carp-specific taqman probe (sequence-6FAM- CCATGTCCGTGAGATTCCAAGCC-TAMRA) designed within the silver carp D-loop region (GenBank: AB595924.1). Thermal cycling conditions were: 95°C for 2 minutes followed by 45 cycles of 95°C for 10 seconds, 58°C for 15 seconds, 61°C for 15 seconds, and a final extension at 61°C for 5 minutes before holding at 4°C. Reactions (20 µL) contained 1 µL of template DNA, 1 µM forward and reverse primers, 50 nM probe, and 10 µL of 2× SensiFAST Probe No-ROX Mix (Bioline USA Inc., Taunton, MA). Each qPCR was done in duplicate, and each plate had additional no template controls and a 7-point calibration curve with plasmid DNA standards of 10^6^, 10^5^, 10^4^, 10^3^, 10^2^, 10, and 0 copies per reaction. The plasmid used contained the cloned D-loop region of silver carp (GenBank: AB595924.1). Amplification was detected by probe fluorescence at 520 nm, and DNA counts were calculated by Mastercycler ep Realplex software with automatic detection of the baseline and threshold setting above noiseband (version 2.2).

### Data Analysis

For all samples, positive detection was noted if a single PCR replicate produced a 191-bp amplicon. Incidence was calculated as the proportion of total samples for each day that had positive detection. Template starting copy numbers were determined by qPCR on 1 µL samples then multiplied by 4,000 (200 µL elution volume from 50 mL samples collected) to calculate copies per liter of water run across the carcasses. The standard error and mean copies/L were calculated across all samples for each day. Results from the 10-carcass chambers and the 1-carcass chambers were compared with Fisher's exact test (presence/absence assays) and Welch's two sample t-test (qPCR assays) using R (version 3.0.0).

## Results

### Persistence of DNA

Silver carp DNA was consistently detected from swabs collected from carcasses, while slime was found to be a variable source of DNA. The DNA of silver carp was detected in all samples taken from carcasses on Day 1 and Day 28. All samples of silver carp slime adhering to metal trays taken on Day 1 and Day 4 contained silver carp DNA. Detection of silver carp DNA in samples of silver carp slime adhering to metal trays was variable from Day 6 through Day 28 as shown in table 1. No correlation was found between detections and weather conditions including maximum temperature, minimum temperature, average humidity, precipitation, and UV index (Table S1).

**Table 1:** Detection of silver carp DNA from slime adhering to metal trays.

Tray	Replicate Swab	Study Day									
		1	4	6	8	10	12	15	17	19	28
**1**	**Rep 1**	+	NA	NA	***(−)***	+	+	+	***(−)***	***(−)***	***(−)***
	**Rep 2**	+	+	+	***(−)***	+	+	+	***(−)***	***(−)***	+
	**Rep 3**	+	+	***(−)***	***(−)***	+	***(−)***	+	***(−)***	***(−)***	+
**2**	**Rep 1**	+	NA	NA	***(−)***	+	+	***(−)***	***(−)***	***(−)***	+
	**Rep 2**	+	+	***(−)***	***(−)***	+	+	***(−)***	***(−)***	***(−)***	***(−)***
	**Rep 3**	+	+	***(−)***	***(−)***	+	+	+	+	***(−)***	***(−)***
**3**	**Rep 1**	+	NA	NA	***(−)***	+	+	+	+	***(−)***	+
	**Rep 2**	+	+	+	+	+	+	+	+	***(−)***	+
	**Rep 3**	+	+	***(−)***	***(−)***	+	+	+	***(−)***	+	+
**4**	**Rep 1**	+	NA	NA	***(−)***	+	***(−)***	+	+	***(−)***	***(−)***
	**Rep 2**	+	+	***(−)***	***(−)***	+	***(−)***	***(−)***	+	***(−)***	***(−)***
	**Rep 3**	+	+	***(−)***	***(−)***	+	***(−)***	+	+	***(−)***	***(−)***

A plus (+) indicates a positive detection of silver carp DNA over time among triplicate swabs from silver carp slime accumulated on metal trays. A minus (−) indicates no detection of silver carp DNA. NA indicates discarded data due to contamination detected in extraction negative controls.

Silver carp DNA was detected in the feces of eagles. All samples collected through Day 18 were positive for silver carp DNA. All fecal samples taken on Day 30 were determined positive for SVC DNA, however 1 runoff sample had only 1 positive aliquot of the 3 taken. The maximum temperature recorded on the surface of the metal trays was 62.2°C on day 12 (Table S2). The harshest weather conditions were recorded on day 0 as 33.9°C maximum ambient temperature with 75% average humidity and a UV index of 9 (Table S3).

### Shedding of DNA from carcasses in water

Silver carp DNA was detected in water samples taken from the chambers containing 10 carcasses throughout the study period. Silver carp DNA was detected in all samples from chambers containing a single fish on Day 1, 4 and 6. The incidence of DNA detection decreased over the study period as shown in table 2.

**Table 2:** Detection of silver carp DNA from water in contact with a silver carp carcass.

Chamber	Replicate Sample	Study Day									
		1	4	6	8	10	12	15	17	19	28
**1**	**Rep 1**	+	+	+	+	***(−)***	***(−)***	***(−)***	***(−)***	***(−)***	***(−)***
	**Rep 2**	+	+	+	***(−)***	+	***(−)***	***(−)***	***(−)***	***(−)***	***(−)***
	**Rep 3**	+	+	+	+	***(−)***	***(−)***	***(−)***	***(−)***	***(−)***	***(−)***
**2**	**Rep 1**	+	+	+	+	+	***(−)***	***(−)***	+	***(−)***	+
	**Rep 2**	+	+	+	+	+	+	+	+	+	***(−)***
	**Rep 3**	+	+	+	+	+	+	***(−)***	+	+	***(−)***
**3**	**Rep 1**	+	+	+	+	+	+	***(−)***	+	+	***(−)***
	**Rep 2**	+	+	+	+	+	***(−)***	***(−)***	***(−)***	***(−)***	***(−)***
	**Rep 3**	+	+	+	+	+	+	***(−)***	+	+	***(−)***
**4**	**Rep 1**	+	+	+	+	+	+	+	+	***(−)***	***(−)***
	**Rep 2**	+	+	+	+	***(−)***	+	+	+	***(−)***	***(−)***
	**Rep 3**	+	+	+	+	+	+	+	+	***(−)***	+

A plus (+) indicates a positive detection of silver carp DNA over time among triplicate water samples from chambers containing a juvenile silver carp carcass. A minus (−) indicates no detection of silver carp DNA.

**Figure 2:**
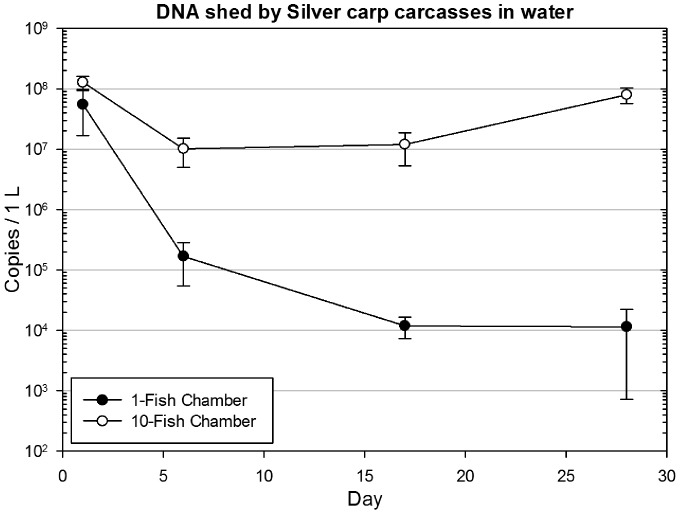
Copies of DNA shed by silver carp carcasses per liter of flowing water. Plots with open circles represent chambers containing 10 juvenile silver carp and plots with dark circles represent chambers containing a single juvenile silver carp. Each point represents the mean of 12 replicate samples (4 replicate chambers, 3 water samples each), and error bars are ± SEM. Copy numbers of the water samples from single-carcass chambers were compared to those from 10-carcass chambers Welch's t-test. On day 28, the water samples from 10-carcass chambers contained significantly more copies of DNA than those from single-carcass chambers (p<0.05).

We found that one or ten carcasses shed an average of 55 million or 128 million copies per liter of water run across them respectively on day one. By day 28, they were still shedding an average of 10,000 and 22 million copies per liter, respectively (Figure 2). Silver carp DNA remained detectable in a higher proportion of samples up to 28 days in the chambers holding 10 carcasses compared to those holding only one (p<0.01). Additionally, the chambers holding 10 carcasses were shedding significantly more detectable DNA than the single carcass chambers at 28 days (p<0.05).

## Discussion

Techniques for isolating and identifying eDNA are an effective and efficient way to detect species presence in freshwater [4]. However, interpreting results may not be as straightforward as previously thought. We found that the DNA of silver carp from carcasses, slime and in avian excrement can be detected by the markers presently used in field surveillance for this species. This detection persisted for ≥28 days. It has been reported by others that DNA can persist in water for days to a month after being shed into the environment [29], [30]. It has been reported that sturgeon DNA persisted in 4.8-m^3^ ponds for two weeks and bullfrog DNA persisted in 900-mL glass beakers for 25 days [29]. It has also been reported that plasmid DNA (pEGFP from Clontech, USA) persisted in surface lake water for 75 hours but degraded little or not at all in hypolimnion water after a full week, suggesting that water chemistry can vary spatially and have a significant impact on DNA persistence in aquatic environments [30]. Prey DNA has been found to survive gut passage to be excreted hours or even days after ingestion [31]–[33], though once excreted, the persistence of prey DNA has not been well studied. Our study to assess DNA persistence used a conservative experimental design and thus excluded some environmental conditions that may have reduced the reported persistence. We felt this was justified, because there may be other environmental factors that may affect eDNA and cause longer persistence. Combined, these findings suggest that DNA from bigheaded carp carcasses, slime or in avian feces may persist for an additional month or more in some aquatic systems.

Several factors that were excluded from our trial may have impacted the persistence of DNA. Ultraviolet light (UV) causes degradation of DNA through induction of pyrimidine dimerization and to a lesser extent, strand breaks, both of which can prevent PCR amplification. UV exposure can vary significantly with altitude, latitude and cloud cover, and the use of plastic covers to prevent rain from washing away samples in our study may have controlled for this by decreasing sample exposure to UV. While increased UV exposure might have reduced DNA persistence, the use of short sequences (191 bp and 107 bp) drastically reduces the susceptibility of these assays to UV degradation [34]. Microbial composition will likely impact degradation, but will vary from one environment to the next. We used well water rather than lake/river water which would have had a higher microbial level and could have decreased persistence. The obvious accumulation of biofilm in the chambers indicated the presence of substantial microbial activity in the chambers. Conversely, DNA shed in our chambers could also be more susceptible to enzymatic activity, because the presence of clay or organic particles suspended in surface water can extend DNA persistence by protecting the DNA from enzyme activity (reviewed in [35]).

Consistent with others [29], [36], our experiments show that DNA persistence is enhanced by greater source density biomass, evidenced by comparing detection in the chambers holding a single carcass to those holding 10 carcasses. This is also consistent with the observation that greater bullfrog densities in wetlands lead to higher rates of detection in [18] and the observation that eDNA concentration is correlated with common carp biomass in [37].

The variable result found with the slime adhering to metal trays (Table 1), particularly the result of only 1 DNA detection among 12 replicates on day 8 followed by 12 DNA detections among 12 replicates on day 10, is indicative of the variable nature of eDNA sampling. The part of the slime deposits swabbed on day 8 may have contained few if any intact cells or otherwise protected DNA, whereas the slime swabbed on day 10 may have been a more DNA dense part of the deposits. Whether swabbing a slime deposit or collecting water, it is unlikely to capture representative DNA for all organisms present in any one sample. Though greater density of the species of interest increases the likelihood of detection, repeated sampling is needed for greater certainty [38].

We show that carcasses, slime residues, and predator feces are all feasible pathways for silver carp DNA to enter the environment. For each transmission pathway, silver carp DNA remained detectable for at least 28 days. These results may confound interpretation of eDNA detection, especially in systems where carcasses, slime and bird feces can regularly be transported from bigheaded carp-infested sites to bigheaded carp-free sites being monitored. As research progresses to refine eDNA detection methodologies, efforts should focus on the development of methods to reduce the potential of vectors to confound eDNA surveillance. There is potential to discover new markers and strategies that may only detect DNA from live fish or recently deceased fish. Another approach of monitoring for environmental RNA, which is typically degraded more rapidly, may be better at detection of recent presence of a live aquatic invasive species than eDNA. Planning sampling strategies that recognize the risk posed by vectors of eDNA (e.g. barge traffic, roosting areas of fish-eating birds, etc.) and focusing sampling in areas where fish are likely present (e.g. spawning sites) could improve the probability of detecting the DNA of live fish. Statistical modeling of eDNA movement and persistence could aid in determining the best sampling strategies. Regardless, further research to optimize sampling strategies and differentiating eDNA from live and remnant sources is vital for improving interpretation of eDNA data.

Our studies show that DNA of silver carp can be detected from multiple transmission pathways independent of the presence of live fish, and this DNA can be detected for more than 28 days. This persistence of DNA suggests the DNA could be transported long distances from its fish source. We have shown that DNA can be detected using markers less than 200 bp well after it has been deposited or shed or after the animal has died. This information will help in the design of future eDNA monitoring programs for improved interpretation of data by natural resource managers.

## Supporting Information

Table S1 **Weather conditions experienced by carcasses and slime on simulated barges.** Table showing the weather conditions as recorded by the NOAA weather station located at the La Crosse, WI airport. UV index was taken from the NOAA/EPA forecast bulletin for Milwaukee, WI.
(DOCX)

Table S2 **Midday temperatures experienced by eagle feces on simulated barges.** Table showing the temperatures (°C) of the surface of the metal trays and the water of the ponds on which they were floating at the time samples were taken. Tray and pond numbers correspond to individual eagles from which fecal matter was collected.
(DOCX)

Table S3 **Weather conditions experienced by eagle feces on simulated barges.** Table showing the weather conditions as recorded by the NOAA weather station located at the La Crosse, WI airport. UV index was taken from the NOAA/EPA forecast bulletin for Milwaukee, WI.
(DOCX)
