# Supplementary material for: Persistence of DNA in Carcasses, Slime and Avian Feces May Affect Interpretation of Environmental DNA Data
Source: PLoS One. 2014 Nov 17;9(11):e113346. doi: 10.1371/journal.pone.0113346 (PMC4234652; doi:10.1371/journal.pone.0113346)
Supplement: Table S2 — Midday temperatures experienced by eagle feces on simulated barges. Table showing the temperatures (°C) of the surface of the metal trays and the water of the ponds on which they were floating at the time samples were taken. Tray and pond numbers correspond to individual eagles from which fecal matter was collected. (DOCX) [file pone.0113346.s002.docx]

|  |  | Pond Rain | | | | No Rain | | | | Water Temperatures | | | |
| --- | --- | --- | --- | --- | --- | --- | --- | --- | --- | --- | --- | --- | --- |
| Date | Day | Tray 1a | Tray 2a | Tray 3a | Tray 4a | Tray 1b | Tray 2b | Tray 3b | Tray 4b | Pond 1a | Pond 2a | Pond 3a | Pond 4a |
| 7/30/2012 | 0 | 46.1 | 47.2 | 42.6 | 47 | 44.4 | 49.8 | 54.4 | 52.6 | 25.1 | 24.5 | 27.6 | 24.8 |
| 7/31/2012 | 1 | 42.6 | 43.6 | 43.4 | 44.9 | 43.9 | 49.9 | 49.3 | 53.6 | 23.4 | 23.4 | 23 | 23.4 |
| 8/1/2012 | 2 | 44.8 | 46.6 | 42.9 | 42.3 | 44.1 | 50 | 48.8 | 50.2 | 25.3 | 24.9 | 24.3 | 24.9 |
| 8/2/2012 | 3 | 44 | 48.1 | 43.9 | 46.4 | 46.7 | 51.3 | 52.4 | 55.6 | 23.2 | 23.7 | 23.8 | 25.3 |
| 8/3/2012 | 4 | 49.6 | 51.9 | 51.7 | 50.5 | 49.8 | 57.5 | 57.6 | 58.2 | 26.6 | 25.8 | 27.2 | 26.4 |
| 8/4/2012 | 5 | 23.7 | 25.4 | 24.6 | 24.6 | 24.5 | 25.8 | 25.4 | 24.9 | 19.8 | 19.4 | 19 | 19.1 |
| 8/5/2012 | 6 | 18.6 | 18.1 | 16.6 | 16.6 | 18.6 | 18.4 | 16.6 | 16.7 | 14.1 | 15.5 | 13.9 | 13.2 |
| 8/6/2012 | 7 | 46.8 | 47.3 | 44.4 | 43.9 | 48.7 | 55.8 | 58.4 | 58.5 | 22.1 | 21.3 | 20.7 | 21.2 |
| 8/7/2012 | 8 | 49 | 53.4 | 52.4 | 52.4 | 56 | 59.8 | 52.8 | 56.9 | 27.6 | 26.6 | 26.5 | 27.3 |
| 8/8/2012 | 9 | 24.5 | 26.3 | 26.3 | 25.6 | 25.7 | 26.2 | 27.3 | 26.5 | 19.4 | 18.9 | 18.9 | 18.8 |
| 8/9/2012 | 10 | 22.3 | 23.9 | 23.3 | 23.8 | 24.3 | 25.9 | 25.6 | 25.6 | 18.2 | 17.9 | 17.6 | 17.5 |
| 8/10/2012 | 11 | 41.8 | 45.2 | 42.3 | 43.1 | 48.2 | 53.2 | 55.9 | 57.4 | 21.5 | 21.8 | 21.2 | 21.6 |
| 8/11/2012 | 12 | 44.8 | 44 | 38.6 | 50.6 | 41.9 | 51 | 52.9 | 54.3 | 21.9 | 21.2 | 22.1 | 22.8 |
| 8/12/2012 | 13 | 28.6 | 30.8 | 25.7 | 31.9 | 29.1 | 29.9 | 32 | 31.6 | 17.9 | 18.2 | 18.4 | 18.1 |
| 8/13/2012 | 14 | 19.2 | 21.1 | 20.3 | 20.7 | 20.9 | 21.9 | 21.1 | 22.6 | 16.1 | 15.2 | 15.1 | 15.5 |
| 8/14/2012 | 15 | 18.4 | 18.8 | 19.1 | 19.2 | 19.1 | 19.3 | 19.9 | 18.9 | 16.6 | 16.3 | 16.4 | 16.5 |
| 8/15/2012 | 16 | 21.9 | 22.8 | 22.7 | 22.1 | 24.1 | 24.2 | 23.7 | 22.3 | 17.9 | 16.8 | 16.4 | 17.1 |
| 8/16/2012 | 17 | 25.4 | 27.9 | 26.1 | 29.1 | 28.6 | 31.8 | 34.3 | 30.9 | 17.6 | 16.7 | 16.8 | 16.3 |
| 8/17/2012 | 18 | 31.3 | 29.6 | 25.6 | 33.3 | 30 | 39.6 | 38.8 | 36.4 | 16.4 | 16.1 | 15.7 | 14.8 |
| 8/29/2012 | 30 | 32.8 | 37.6 | 32.3 | 31.9 | 32.4 | 34.9 | 32.4 | 35 | 21.1 | 19.8 | 19.4 | 19.8 |
|  |  | DI Rain | | | | No Rain | | | | Water Temperatures | | | |
| Date | Day | Tray 1c | Tray 2c | Tray 3c | Tray 4c | Tray 1d | Tray 2d | Tray 3d | Tray 4d | Pond 1c | Pond 2c | Pond 3c | Pond 4c |
| 7/30/2012 | 0 | 45.4 | 50.3 | 50.4 | 52.5 | 51.6 | 53.8 | 52.9 | 51.3 | 26 | 25.6 | 25.4 | 25.6 |
| 7/31/2012 | 1 | 43.1 | 41.9 | 45.6 | 52.3 | 52.9 | 44.6 | 47 | 51.3 | 24.5 | 23.4 | 24.3 | 23.9 |
| 8/1/2012 | 2 | 40.6 | 47.9 | 43.4 | 46.2 | 48.9 | 47.4 | 42.9 | 46.9 | 24.4 | 24.3 | 24.2 | 23.9 |
| 8/2/2012 | 3 | 34.3 | 43.6 | 39.6 | 44.9 | 37.3 | 39.5 | 38.5 | 41.3 | 22.2 | 22.8 | 22.6 | 22.6 |
| 8/3/2012 | 4 | 46.38 | 55.6 | 51.2 | 57.7 | 55.3 | 54.1 | 50.7 | 55.3 | 24.1 | 25.1 | 25.2 | 24.6 |
| 8/4/2012 | 5 | 23.6 | 23.8 | 24 | 22.6 | 24.8 | 23.8 | 24 | 23.6 | 20.6 | 20.6 | 20.7 | 20.9 |
| 8/5/2012 | 6 | 20 | 21.5 | 20.6 | 18.6 | 20.7 | 19.9 | 21 | 17.1 | 15.5 | 15.4 | 15.1 | 15 |
| 8/6/2012 | 7 | 44.1 | 52.1 | 48.6 | 48.4 | 57.8 | 54.1 | 50.4 | 54.8 | 26.1 | 22.3 | 21.5 | 21.3 |
| 8/7/2012 | 8 | 45.4 | 53.7 | 48.5 | 52.4 | 53.3 | 55.4 | 52.8 | 61.3 | 24.1 | 23.8 | 25.5 | 25.6 |
| 8/8/2012 | 9 | 23 | 24.6 | 24.1 | 24.1 | 23.4 | 24.1 | 23.7 | 24.6 | 18.7 | 18.4 | 18.6 | 18.4 |
| 8/9/2012 | 10 | 21.1 | 23.6 | 22.7 | 23.6 | 22.8 | 23.8 | 23.4 | 23.8 | 16.8 | 16.7 | 16.7 | 16.8 |
| 8/10/2012 | 11 | 38.5 | 49.4 | 39.7 | 41.9 | 53.4 | 50.5 | 39.6 | 43.4 | 20.3 | 20.2 | 19.6 | 19.1 |
| 8/11/2012 | 12 | 42.9 | 41.3 | 46.8 | 58.4 | 54.8 | 53.9 | 50.4 | 62.2 | 22.1 | 21.8 | 22.3 | 25 |
| 8/12/2012 | 13 | 25.7 | 22.6 | 24.9 | 22.6 | 27.8 | 28.3 | 29 | 26.7 | 18.3 | 17.8 | 17.4 | 17.7 |
| 8/13/2012 | 14 | 19.9 | 22.2 | 21.1 | 20 | 19.1 | 22.3 | 22.7 | 21.1 | 15.9 | 15.7 | 15.8 | 15.6 |
| 8/14/2012 | 15 | 18.6 | 19.3 | 18.9 | 19.1 | 19.4 | 19.4 | 19.3 | 19.2 | 17.2 | 16.9 | 16.8 | 16.8 |
| 8/15/2012 | 16 | 26 | 27.3 | 27 | 26.1 | 25.3 | 28.3 | 27.3 | 21.9 | 17.2 | 17 | 17 | 17.2 |
| 8/16/2012 | 17 | 25.6 | 27.5 | 27.6 | 29.6 | 29.4 | 32.1 | 30.7 | 30.3 | 18.2 | 18 | 17.8 | 18.1 |
| 8/17/2012 | 18 | 28.6 | 35.1 | 32.9 | 41.8 | 38.9 | 39.9 | 41.1 | 42.8 | 17.4 | 17.2 | 15.2 | 17.2 |
| 8/29/2012 | 30 | 32.6 | 34.9 | 33.2 | 32.9 | 33.7 | 34.6 | 33.7 | 35.7 | 19.9 | 19.8 | 19.8 | 19.3 |
