# Supplementary material for: Persistence of DNA in Carcasses, Slime and Avian Feces May Affect Interpretation of Environmental DNA Data
Source: PLoS One. 2014 Nov 17;9(11):e113346. doi: 10.1371/journal.pone.0113346 (PMC4234652; doi:10.1371/journal.pone.0113346)
Supplement: Table S3 — Weather conditions experienced by eagle feces on simulated barges. Table showing the weather conditions as recorded by the NOAA weather station located at the La Crosse, WI airport. UV index was taken from the NOAA/EPA forecast bulletin for Milwaukee, WI. (DOCX) [file pone.0113346.s003.docx]

| Date | Day | Max Temp (°C) | Min Temp (°C) | Average Humidity | Rain (mm) | UV Index |
| --- | --- | --- | --- | --- | --- | --- |
| 7/30/2012 | 0 | 33.9 | 17.2 | 75% | 0.00 | 9 |
| 7/31/2012 | 1 | 30.6 | 17.8 | 66% | 0.00 | 8 |
| 8/1/2012 | 2 | 33.9 | 15.6 | 71% | 0.00 | 8 |
| 8/2/2012 | 3 | 30.0 | 20.0 | 69% | 3.81 | 7 |
| 8/3/2012 | 4 | 33.9 | 17.8 | 68% | 0.00 | 8 |
| 8/4/2012 | 5 | 26.7 | 18.3 | 75% | 33.53 | 8 |
| 8/5/2012 | 6 | 24.4 | 15.0 | 63% | 0.00 | 8 |
| 8/6/2012 | 7 | 28.3 | 13.3 | 70% | 0.00 | 8 |
| 8/7/2012 | 8 | 32.2 | 17.8 | 64% | 0.00 | 8 |
| 8/8/2012 | 9 | 26.7 | 15.6 | 78% | 1.02 | 7 |
| 8/9/2012 | 10 | 20.6 | 13.9 | 80% | 0.00 | 7 |
| 8/10/2012 | 11 | 25.0 | 13.9 | 66% | 0.00 | 5 |
| 8/11/2012 | 12 | 25.6 | 9.4 | 64% | 0.00 | 7 |
| 8/12/2012 | 13 | 21.7 | 15.6 | 77% | 1.02 | 7 |
| 8/13/2012 | 14 | 27.2 | 15.6 | 73% | 0.76 | 1 |
| 8/14/2012 | 15 | 27.2 | 15.6 | 71% | 1.02 | 7 |
| 8/15/2012 | 16 | 28.9 | 16.7 | 67% | 0.25 | 7 |
| 8/16/2012 | 17 | 23.3 | 13.3 | 77% | 3.81 | 3 |
| 8/17/2012 | 18 | 22.8 | 10.0 | 67% | 0.00 | 7 |
| 8/29/2012 | 30 | 33.3 | 18.3 | 63% | 0.00 | 7 |
